# Role perceptions and experiences of adult children in remote glucose management for older parents with type 2 diabetes mellitus: a qualitative study

**DOI:** 10.1186/s12877-024-05224-6

**Published:** 2024-08-03

**Authors:** Xiang Ye, Rongzhen Liu, Shangjie Che, Yanqun Zhang, Jiaqi Wu, Ya Jiang, Xiangrong Luo, Cuihua Xie

**Affiliations:** 1 Department of Endocrinology and Metabolism, Nanfang Hospital, Southern Medical University, No.1838, Guangzhou Avenue North, Baiyun District, Guangzhou, China; 2 School of Nursing, Southern Medical University, Guangzhou, China; 3 Department of Emergency, Nanfang Hospital, Southern Medical University, No.1838, Guangzhou Avenue North, Baiyun District, Guangzhou, China

**Keywords:** Adult children, Glucose management, Older parents, Type 2 diabetes mellitus, Qualitative study, Mobile app

## Abstract

**Background:**

With the advent of the smart phone era, managing blood glucose at home through apps will become more common for older individuals with diabetes. Adult children play important roles in glucose management of older parents. Few studies have explored how adult children really feel about engaging in the glucose management of their older parents with type 2 diabetes mellitus (T2DM) through mobile apps. This study provides insights into the role perceptions and experiences of adult children of older parents with T2DM participating in glucose management through mobile apps.

**Methods:**

In this qualitative study, 16 adult children of older parents with T2DM, who had used mobile apps to manage blood glucose for 6 months, were recruited through purposive sampling. Semi-structured, in-depth, face-to-face interviews to explore their role perceptions and experiences in remotely managing their older parents’ blood glucose were conducted. The Consolidated Criteria for Reporting Qualitative Research (COREQ) were followed to ensure rigor in the study. The data collected were analyzed by applying Colaizzi’s seven-step qualitative analysis method.

**Results:**

Six themes and eight sub-themes were identified in this study. Adult children’s perceived roles in glucose management of older parents with T2DM through mobile apps could be categorized into four themes: health decision-maker, remote supervisor, health educator and emotional supporter. The experiences of participation could be categorized into two themes: facilitators to participation and barriers to participation.

**Conclusion:**

Some barriers existed for adult children of older parents with T2DM participating in glucose management through mobile apps; however, the findings of this study were generally positive. It was beneficial and feasible for adult children to co-manage the blood glucose of older parents. Co-managing blood glucose levels in older parents with T2DM can enhance both adherence rates and confidence in managing blood glucose effectively.

**Supplementary Information:**

The online version contains supplementary material available at 10.1186/s12877-024-05224-6.

## Background

According to the latest data from the International Diabetes Federation, the global prevalence of T2DM in adults was 537 million people in 2021, and a projected 783 million people will be living with diabetes by 2045 [1]. Surveys from 2015 to 2017 showed that the prevalence of diabetes in China was above 20% in older adults aged 60 years and over [2]. According to data from China’s National Bureau of Statistics, 190 million people aged 65 years and over were living with diabetes in China by the end of 2020, accounting for 13.5% of the total population [3]. Population aging has become a major issue, challenging the sustainability of healthcare and social care services.

Mobile apps are effective tools for managing blood glucose, diet, activity and wounds [4–7]. Older adults as a target group may benefit from using apps [8]. Nevertheless, older adults face many barriers in using technology for healthcare decision-making, including issues with familiarity, willingness to ask for help, trust of the technology, privacy and design challenges [9]. Older adults may need support and assistance from their family members when they use mobile apps, and family support is mostly from their spouses and children [10]. Studies are lacking on how adult children really feel about engaging in the glucose management of their older parents with T2DM through mobile apps. Therefore, this study aimed to explore the role perceptions and experiences of adult children involved in remote glucose management of older parents with T2DM.

## Methods

### Study design

This qualitative study used a phenomenological approach involving a series of personal, semi-structured interviews. This study was conducted based on the COREQ [11]. It was conducted from May 2022 to February 2023 at the Department of Endocrinology and Metabolism of a tertiary care hospital in Guangzhou, China.

### Recruitment and participants

To capture diverse insights relevant to our research theme, purposive sampling was employed. The inclusion criteria were as follows: ① participants with a father/mother aged ≥ 65 years diagnosed with type 2 diabetes [12]; ② participants aged ≥ 18 years and <65 years; ③ followed the app of Sinomedisite Glucose Manager via WeChat and checked father’s/mother’s blood glucose data at least once a week for ≥ 6 months; ④ no reading and communication disorders. Exclusion criteria included individuals with cognitive impairment, psychiatric disorders (such as depression, anxiety disorders, schizophrenia, and bipolar disorders), hearing impairments, or those who were unable to independently communicate verbally in Mandarin. Sample size was based on data saturation. Each participant was provided with a glucometer and complimentary access to the management app. Ethical approval for this study was obtained from the ethical committees of Nanfang Hospital of Southern Medical University (NFSC-2022-002) and written informed consent was provided by all participants.

### App description

The app of Sinomedisite Glucose Manager has the following features:

1. Automatic uploading of blood glucose data: The blood glucose data can be automatically uploaded through the wireless network.
2. Data recording: Parents can record their diet, exercise, etc. in the APP.
3. Data analysis: The APP can intelligently generate blood glucose curves, the average fasting blood glucose, the average postprandial blood glucose, the adherence rate for blood glucose control, the incidence of hyperglycemia and hypoglycemia.
4. Knowledge and video section: The APP is e-Health literacy friendly and can provide information on diabetes through text or video formats.
5. Communication: Parents can send messages to administrator for assistance through the APP.
6. Co-managing blood glucose: Adult children can receive real-time updates of their parents’ blood glucose levels and reminder of abnormal readings through the APP.

### Focus group guide

The final focus group guide (Table 1) used in this study was developed based on the COREQ, literature, discussions within the research team, expert consultations and pre-interviews. To minimize bias, a trained researcher conducted the semi-structured interviews. At the conclusion of each session, interviewees were prompted to add any further comments.

**Table 1:** Focus group guide

1. What is your understanding of adult children co-managing older parents’ blood glucose?
2. What is your role in using the app to co-manage your parent’s blood glucose?
3. Can you talk about your experiences of co-managing blood glucose through the mobile app?
4. What do you think are the factors that affect you to take part in glucose management of your parent through the mobile app?
5. What are the difficulties in using the app? How did you cope with them?
6. What are your comments and suggestions for participating in glucose management via app?

### Data analysis

The data were analyzed using Colaizzi’s seven-step qualitative analysis method. The transcription and analysis processes were conducted concurrently by 2 trained reviewers. The audio recordings were transcribed into text within 24 h following the interviews, and any ambiguities or uncertainties were clarified or verified by consulting the interviewees. Nvivo12 supported the organization of codes and themes during data analysis. The textual data transcribed from each interview were imported into the Nvivo12 project. After meticulously reviewing the original materials, two independent reviewers extracted key phrases and sentences, coding them individually. Discrepancies were discussed and resolved until a 90% consensus was achieved. Persistent disagreements were adjudicated by an expert in qualitative research, based on the inputs from the two reviewers. The final nodes and themes were agreed upon by the research team to reduce bias and verified by re-engaging with the interviewees. After data analysis, representative quotes for each theme and sub-theme are selected and presented in Supplementary file 1.

## Results

### Demographic data

After interviewing 14 patients, no new themes emerged in the last two interviews, indicating that data saturation had been reached. Hence, we stopped the interviews. Sixteen face-to-face, semi-structured interviews were conducted in a separate examination room in the department, each lasting approximately 15–40 min, which were audio-recorded with participants consent. Demographic characteristics of interviewees are shown in Table 2.

**Table 2:** Demographic characteristics of interviewees

Variable	Frequency (%)
Interviewees’ characteristics (*n* = 16)	
Gender	
Men	7 (43.8)
Women	9 (56.2)
Occupational status	
Employed	15 (93.8)
Unemployed	1 (6.2)
Education	
Junior diploma or below	4 (25.0)
Above Junior diploma	12(75.0)
Living with patients	
NO	12(75.0)
YES	4(25.0)
Know blood glucose control goals	
NO	6(37.5)
YES	10(62.5)
Family monthly income (CNY ^a^ )	
≤ 10,000	5 (31.3)
>10,000	11 (68.7)

^a^ CNY is Chinese Yuan

### Themes

Among adult children’s role perceptions, the following four themes were identified: (1) health decision-maker; (2) remote supervisor; (3) health educator; and (4) emotional supporter. Among adult children’s experiences of participation, the following two themes were identified: (1) Facilitators to participation; and (2) Barriers to participation. The details of these themes are shown in Table 3.

**Table 3:** Study themes and sub-themes

	Themes	Sub-themes
Role perceptions	Health decision-maker	
	Remote supervisor	
	Health educator	
	Emotional supporter	
Experiences of participation	Facilitators to participation	Convenience
		App user-friendly
		Digital empowerment
		Family-based health promotion
	Barriers to participation	Privacy and security concerns
		Adaptation and learning
		Lack of knowledge about glucose management
		Concerns oven the cost of test strips

Role perceptions of adult children co-managing blood glucose for older parents with T2DM using a mobile app

1. *Health decision-maker* Older parents with T2DM often experience a higher prevalence of cognitive dysfunction, which may impair their ability to make timely and appropriate health decisions. Adult children can keep track of their parents’ blood glucose through the mobile app. Information sharing can help adult children provide timely and appropriate decision-making for older parents with T2DM.

*N7: “As soon as I received the message that his blood sugar was 3.2 mmol/L*,* I called him to drink sugar water and test his blood sugar again 15 minutes later. I reduced his bedtime insulin by 4 units after consulting with the doctor. After that*,* he rarely suffered from hypoglycemia.”*

*N15: “The mobile app revealed that the adherence rate for blood sugar control was only 50%. The reason for blood sugar levels not meeting the standard may be related to excessive rice consumption. Therefore*,* I suggested she measure postprandial blood sugar*,* which was 16 mmol/L. Following the dietitian’s advice*,* I recommended she limit her rice intake to 75 g per meal. As she was unsure how to measure the rice*,* I bought an electronic scale and taught her how to use it to control her staple food intake. Consequently*,* her adherence rate improved from 50–60%.”*

2. *Remote supervisor* Some interviewees said that they were far away from their parents. By viewing their parents’ blood glucose data through the mobile app, they could track blood glucose data and remind their parents to address abnormal blood glucose levels by taking medication and adjusting their diet when experiencing irregular readings.

*N7: “Although my father doesn’t live with me*,* I can keep an eye on his blood sugar through the mobile app. When his blood sugar is lower than 5.6 mmol/L*,* I remind him to eat 3 pieces of soda crackers or drink a glass of milk.”*

*N9: “As my father is old and has a bad memory*,* he would miss his medication. I checked to see if his blood sugar was up to standard via the mobile app and reminded him daily not to miss his medication.”*

3. *Health educators* Adult children can give timely guidance to their parents when they see abnormal blood glucose data via the mobile app, breaking the constraints of time and space, and helping their parents adopt behaviors conducive to optimizing blood glucose.

*N2: “I can check his blood sugar data anytime and anywhere through the app. One day he ate plain congee resulting in high blood sugar. Then I told him not to drink plain congee. If he wants it*,* he should add beans*,* wheat*,* and lean meat to plain congee.”*

*N4: “ I received a message which showed that she suffered from hypoglycemia. It turned out that exercising on an empty stomach caused her hypoglycemia. I stressed the precautions to be taken in exercising. After that*,* she never exercises on an empty stomach.”*

4. *Emotional supporter* older individuals with diabetes are prone to psychological problems such as depression, anxiety and fear because of the long duration of the disease, complications, hypoglycemia and other unexpected situations. Adult children can provide emotional support to parents in time, alleviating their negative emotions and increasing their confidence in glucose control.

*N1: “I saw his blood sugar fluctuating wildly via the mobile app and called him to ask why. He said he had poor sleep and wondered if he was suffering from depression. I comforted him that everything would be fine. After two weeks of adjustments*,* his mood was improved.”*

*N6: “My mother had anxiety and was terrified of hypoglycemia. I told her to take it easy and monitor her blood sugar regularly so that I could keep track of her blood sugar. I taught her ways to cope with hypoglycemia. She’s not so scared anymore and confident in her blood sugar control.”*

The experiences of participation in the glucose management for older parents with T2DM using a mobile app

1. *Facilitators to participation* There are facilitators to participation, such as convenience, app user-friendly, digital empowerment and family-based health promotion.

1.1 *Convenience* Adult children can access blood glucose data online, eliminating the need to look through manual records.

*N7: “Although my father doesn’t live with me*,* I can keep an eye on his blood sugar anytime and anywhere through the mobile app.”*

*N8: “I used to have to keep track of his blood sugar through his paper records. But now I can check his blood sugar through the app. It’s very convenient.”*

1.2 *App user-friendly* The blood glucose data are presented in a visual curve, which makes management more intuitive and accurate. The app can display the adherence rate for blood glucose control and the incidence of hyperglycemia and hypoglycemia in a variety of graphical outputs.

*N1:“The blood sugar data are presented in a visual curve*,* which makes management more intuitive and accurate. So I find it very*,* very useful.”*

*N12: “Information collected can be presented in a variety of graphical outputs. It’s so easy and intuitive now that I no longer worry her about misremembering or missing records.”*

1.3 *Digital empowerment* Digital blood glucose management enables adult children to use the mobile app to improve the ability and efficiency of their older parents with T2DM to manage blood glucose levels, instead of relying solely on healthcare professionals.

*N6: “From the time I told her blood sugar control goals and taught her how to check her adherence rate via the mobile app*,* she paid more attention to her blood sugar and monitored blood sugar regularly.”*

*N14: “I learned that exercising after meal can lower postprandial blood sugar through the app. Based on her exercise and blood sugar*,* I worked with her on an exercise programme which increased her adherence rate for blood sugar control to 70%.”*

1.4 *Family-based health promotion* Engagement of adult children in managing the blood glucose levels of their older parents with T2DM enhances familial support. This includes aiding in dietary choices and financing treatment, thereby improving glucose control and promoting health.

*N8: “He has diabetic nephropathy. In order to control his protein intake and blood sugar*,* I help him prepare breakfast*,* lunch and dinner. The mobile app showed that his adherence rate has increased from 50–80%.”*

*N13: “I and my sister kept track of her blood sugar via the app and bought her medication monthly. After a period of medication*,* her blood sugar was under control.”*

2. *Barriers to participation* There are barriers to participation, such as privacy and security concerns, adaptation and learning, lack of knowledge about glucose management, and concerns oven the cost of test strips.

2.1 *Privacy and security concerns* A few interviewees stated that they had concerns about entering personal information into a mobile app owing to the fear of security breaches.

*N5: “While we can help the elderly manage their blood sugar through mobile apps*,* I am worried that my mother’s information will be leaked.”*

*N7: “Nowadays there are a lot of scammers. The elderly are easily deceived. I am afraid that the personal information which is entered into the app will be utilized by an illegal actor after it is leaked.”*

2.2 *Adaptation and learning* It takes time to learn and get used to the new application.

*N8: “I wasn’t used to use the app at first.”*

*N11: “After 2 weeks of adaptation and consulting with doctors and nurses*,* I am now proficient in using the mobile app.”*

2.3 *Lack of knowledge about glucose management* Lack of knowledge may affect adult children to take part in glucose management of older parents.

*N3:“I didn’t know his blood sugar control goals. There’s no point in focusing on his blood sugar via app.”*

*N11: “One day his blood sugar was 3.8 mmol/L. I was afraid that he would have hypoglycemic symptoms and told him not to take his pre-breakfast insulin injection. As a result*,* his blood sugar was 18 mmol/L in the 2 h after the meal. When an emergency occurs*,* I still don’t know what to do.”*

2.4 *Concerns oven the cost of test strips* Several adult children expressed concerns regarding the high cost of test strips, which were perceived as unaffordable. This financial burden could deter both older parents and their children from purchasing these essential supplies, resulting in less frequent blood glucose monitoring by the older parents. Consequently, this infrequent monitoring could adversely affect the reliability of the glucose data accessed via apps by adult children, diminishing their motivation and efficacy in jointly managing their older parents’ condition.

*N4: “The test strips are too expensive. I have already bought her test strips twice*,* which were not reimbursable.”*

*N9:“Do you provide complimentary test strips? He hesitates to incur expenses*,* thus rarely monitors his blood sugar. Consequently*,* I can access only limited data regarding his blood sugar levels.”*

*N16: “The test strips are so expensive that I can’t afford them.”*

## Discussion

The current qualitative study provided insight into the role perceptions and experiences of adult children of older parents with T2DM in participating in glucose management through a mobile app. Six themes and eight sub-themes were identified in this study.

Family-involvement intervention is helpful in diabetes management [13]. Higher levels of social support are often associated with increased disease knowledge, better medication adherence, better self-efficacy and glucose control [13–17]. As important family members of older parents with T2DM, adult children assume an important role in geriatric diabetes care. The results of this study showed that most of adult children were able to provide health decision-making, remote supervision, health education, and emotional support to older parents with T2DM by participating in glucose management through a mobile app.

Significant and positive associations have been found between diabetes and anxiety disorders [18]. Diabetes and depression are frequently comorbid in older adults [19]. Older adults with diabetes have unique psychological and medical challenges that impact self-care and glucose control [20]. Most adult children in this study were able to provide emotional and psychological support for their older parents, which alleviate their negative emotions and increase their confidence in glucose control.

Mobile apps can improve self-management behavior of patients in terms of dietary control, physical exercise, blood glucose monitoring, medication adherence, and screening of complications [17, 21–23]. Studies have shown that mobile apps help individuals with diabetes to control their blood glucose effectively [24–29]. However, older adults face many barriers in using technology for healthcare decision-making, including issues with familiarity, privacy, and design challenges [9]. Therefore, there is a need for adult children to use these mobile apps to assist their older parents in glucose management.

Many studies have reported that issues of privacy and security are a major concern [9, 30], and our study echoed this. These problems are further exacerbated when traditional paper records are transferred to an electronic medium. We have found many important challenges in implementing a secure healthcare monitoring system using medical sensors [31]. A systematic review and meta-analysis indicated that older adults are susceptible to fraud [32]. In this study, some interviewees expressed the concerns about the privacy and security of the mobile app, fearing potential information leak about their parents. In order to protect the security and privacy of older adults, we have enhanced testing and maintenance for the APP and assured that the information will be kept confidential.

Technical barriers can result in decreasing intention to use the app [33]. This study showed that several adult children had difficulties in applying mobile apps and needed time to adapt. Healthcare professionals should instruct adult children of older parents in the use of mobile apps and appraise them if necessary to make sure that they have mastered use of the apps. Barriers to app use include participant’s technological literacy and lack of knowledge and awareness of apps as healthcare tools [34]. So we need to improve the knowledge and awareness of apps as effective tools to control blood glucose. Several interviewees in this study reported that app motivated them to learn about diabetes and improved the ability and efficiency of the older parents to control their blood glucose instead of relying solely on healthcare providers.

This study showed that a small proportion of adult children lacked knowledge about glucose management. Family members involved in a patient’s diabetes management may impede the patient’s self-care and compromise glucose control unless the family members are taught to avoid obstructive behaviors [35]. Non-supportive behaviors include nagging, arguing, getting in the way of patient’s self-care, food temptation, visible irritation, and refusing to share the burden of living with diabetes [36–38]. Non-supportive behaviors among family members are thought to be associated with patients being less adherent to their diabetes medication regimen, and being less adherent is associated with worse glucose control [39]. Family members with knowledge of diabetes can provide supportive behaviors and participate in glucose management of older adults with diabetes. Studies have shown that people with diabetes have significantly higher levels of health literacy and diabetes knowledge when they care for themselves with additional caretaker assistance [14]. Health knowledge can have an impact on the self-management abilities by bolstering individuals’ confidence to modify health behaviors, and has positive implications for health outcomes among older adults residing in social housing [40]. The knowledge of patients and their key supporters should be regularly assessed in future practice. In the development and implementation of telehealth programs, the level of health literacy of caretakers should be fully considered. Furthermore, training programs should be developed for key supporters to improve their knowledge of diabetes.

Adult children’s attention to the blood glucose levels of their parents through mobile apps relies on the patient performing blood glucose monitoring. If older parents with diabetes do not monitor their blood glucose, adult children will not be able to participate in glucose management. Some interviewees in this study indicated that the test strips were so expensive that they could not afford them. This is consistent with a previous study, in which the cost of self-monitoring of blood glucose (SMBG) was the main reason why participants did not practice SMBG regularly [41]. This financial burden could deter both older parents and their children from purchasing these essential supplies, resulting in less frequent blood glucose monitoring by the older parents. The state could reimburse a percentage of the cost of test strips for older individuals with diabetes when developing health insurance policies. Healthcare professionals should set a personalized frequency of SMBG for older people with diabetes.

### Strengths and limitations

The current study had several noteworthy strengths. First, the current study reported according to the COREQ. Second, in order to increase the reliability of the study, any differences in design, methodology, data analysis and results were discussed by the research team until agreement was reached. Third, all data were analyzed by the study team. Interviewees were audio-recorded to ensure the authenticity of the findings and, during the course of the study, the researchers retained the focus group guide, recorded data, original transcriptions, and study results.

Nevertheless, there were limitations to the current study. Interviewees were recruited from a tertiary care hospital. Thus, the sample was not representative of all adult children’s perceptions of co-managing the blood glucose of their older parents with T2DM through a mobile app. Therefore, the findings and conclusions should be interpreted with caution in terms of universality. Future research is needed to (1) evaluate the usability of mobile app in older adults with diabetes and (2) assess the effectiveness of adult children’s involvement in glucose management of older parents with diabetes via mobile app through randomized controlled trials, trials with larger sample sizes, trials with effective recruitment strategies and sampling methods, and trials in different settings such as primary care.

## Conclusion

Although there were barriers to participation in glucose management of older parents with T2DM through a mobile app, the findings of this study were generally positive. It was beneficial and feasible for adult children to co-manage the blood glucose of their older parents. Co-managing blood glucose levels in older parents with T2DM can enhance both adherence rates and confidence in managing blood glucose effectively.

The results of the current study can provide the basis for the development of telemedicine and telehealth in geriatric care, and promote the in-depth application of internet, mobile apps, and other information technologies in elderly services.

### Electronic supplementary material

Below is the link to the electronic supplementary material.

Supplementary Material 1

## Data Availability

The data generated and/or analyzed during the study are not publicly available but are available from the authors upon reasonable request and with permission of the corresponding author.

## Abbreviations

T2DM: Type 2 Diabetes Mellitus
COREQ: Consolidated Criteria for Reporting Qualitative Research
SMBG: Self-Monitoring of Blood Glucose

## References

[1] Magliano DJ, Boyko EJDA. IDF DIABETES ATLAS. Brussels: International Diabetes Federation; 2021.

[2] Li Y, Teng D, Shi X, et al. Prevalence of diabetes recorded in mainland China using 2018 diagnostic criteria from the American Diabetes Association: national cross sectional study. BMJ. 2020;369:m997. 10.1136/bmj.m997. 32345662 10.1136/bmj.m997 PMC7186854

[3] MAJOR FIGURES ON 2020 POPULATION CENSUS OF CHINA. https://www.stats.gov.cn/sj/pcsj/rkpc/d7c/202303/P020230301403217959330.pdf. Accessed 10 Jul 2023.

[4] Quinn CC, Shardell MD, Terrin ML Cluster-randomized trial of a mobile phone personalized behavioral intervention for blood glucose control. DIABETES CARE., Rigla M, Martínez-Sarriegui I, García-Sáez G et al. Gestational Diabetes Management Using Smart Mobile Telemedicine. J Diabetes Sci Technol. 2018;12:260-4. 10.1177/1932296817704442.

[5] Zhang W, Yu Q, Siddiquie B, et al. Snap-n-Eat: Food Recognition and Nutrition Estimation on a smartphone. J Diabetes Sci Technol. 2015;9:525–33. 10.1177/1932296815582222. 25901024 10.1177/1932296815582222 PMC4604540

[6] Cvetković B, Janko V, Romero AE, et al. Activity Recognition for Diabetic patients using a smartphone. J Med Syst. 2016;40:256. 10.1007/s10916-016-0598-y. 27722975 10.1007/s10916-016-0598-y

[7] Wang L, Pedersen PC, Strong DM, et al. Smartphone-based wound assessment system for patients with diabetes. IEEE Trans Biomed Eng. 2015;62:477–88. 10.1109/TBME.2014.2358632. 25248175 10.1109/TBME.2014.2358632

[8] Wong A, Wong F, Bayuo J, et al. A randomized controlled trial of an mHealth application with nursing interaction to promote quality of life among community-dwelling older adults. Front Psychiatry. 2022;13:978416. 10.3389/fpsyt.2022.978416. 36329920 10.3389/fpsyt.2022.978416 PMC9623156

[9] Fischer SH, David D, Crotty BH, et al. Acceptance and use of health information technology by community-dwelling elders. Int J Med Informatics. 2014;83:624–35. 10.1016/j.ijmedinf.2014.06.005. 10.1016/j.ijmedinf.2014.06.005 PMC4144164 24996581

[10] Pesantes MA, Del VA, Diez-Canseco F, et al. Family support and diabetes: patient’s experiences from a Public Hospital in Peru. Qual Health Res. 2018;28:1871–82. 10.1177/1049732318784906. 30066604 10.1177/1049732318784906 PMC6346298

[11] Tong A, Sainsbury P, Craig J. Consolidated criteria for reporting qualitative research (COREQ): a 32-item checklist for interviews and focus groups. Int J Qual Health Care. 2007;19:349–57. 10.1093/intqhc/mzm042. 17872937 10.1093/intqhc/mzm042

[12] 2. Classification and diagnosis of diabetes: standards of Medical Care in Diabetes-2022. Diabetes Care. 2022;45:S17–38. 10.2337/dc22-S002. 34964875 10.2337/dc22-S002

[13] Withidpanyawong U, Lerkiatbundit S, Saengcharoen W. Family-based intervention by pharmacists for type 2 diabetes: a randomised controlled trial. Patient Educ Couns. 2019;102:85–92. 10.1016/j.pec.2018.08.015. 30150128 10.1016/j.pec.2018.08.015

[14] Yeh JZ, Wei CJ, Weng SF, et al. Disease-specific health literacy, disease knowledge, and adherence behavior among patients with type 2 diabetes in Taiwan. BMC Public Health. 2018;18:1062. 10.1186/s12889-018-5972-x. 30143020 10.1186/s12889-018-5972-x PMC6108149

[15] Olagbemide OJ, Omosanya OE, Ayodapo AO, et al. Family support and medication adherence among adult type 2 diabetes: any meeting point ? Ann Afr Med. 2021;20:282–7. 10.4103/aam.aam_62_20. 34893566 10.4103/aam.aam_62_20 PMC8693738

[16] García-Huidobro D, Bittner M, Brahm P, et al. Family intervention to control type 2 diabetes: a controlled clinical trial. Fam Pract. 2011;28:4–11. 10.1093/fampra/cmq069. 20817790 10.1093/fampra/cmq069

[17] Huang Z, Tan E, Lum E, et al. A smartphone app to improve medication adherence in patients with type 2 diabetes in Asia: Feasibility Randomized Controlled Trial. JMIR mHealth uHealth. 2019;7:e14914. 10.2196/14914. 31516127 10.2196/14914 PMC6746066

[18] Smith KJ, Béland M, Clyde M, et al. Association of diabetes with anxiety: a systematic review and meta-analysis. J Psychosom Res. 2013;74:89–99. 10.1016/j.jpsychores.2012.11.013. 23332522 10.1016/j.jpsychores.2012.11.013

[19] Park M, Reynolds CR. Depression among older adults with diabetes mellitus. Clin Geriatr Med. 2015;31:117–37. 10.1016/j.cger.2014.08.022. 25453305 10.1016/j.cger.2014.08.022 PMC4254540

[20] Beverly EA, Ritholz MD, Shepherd C, et al. The Psychosocial challenges and Care of older adults with diabetes: can’t do what I used to do; can’t be who I once was. Curr Diab Rep. 2016;16:48. 10.1007/s11892-016-0741-7. 27085863 10.1007/s11892-016-0741-7 PMC5469362

[21] Pamungkas RA, Usman AM, Chamroonsawasdi K, et al. A smartphone application of diabetes coaching intervention to prevent the onset of complications and to improve diabetes self-management: a randomized control trial. Diabetes Metab Syndr. 2022;16:102537. 10.1016/j.dsx.2022.102537. 35724489 10.1016/j.dsx.2022.102537

[22] Zhai Y, Yu W. A Mobile App for Diabetes Management: impact on self-efficacy among patients with type 2 diabetes at a Community Hospital. Med Sci Monit. 2020;26:e926719. 10.12659/MSM.926719. 33196634 10.12659/MSM.926719 PMC7678242

[23] Clements MA, Staggs VS. A Mobile App for Synchronizing Glucometer Data: impact on adherence and Glycemic Control among youths with type 1 diabetes in Routine Care. J Diabetes Sci Technol. 2017;11:461–7. 10.1177/1932296817691302. 28745097 10.1177/1932296817691302 PMC5505434

[24] Hou C, Carter B, Hewitt J, et al. Do Mobile phone applications improve Glycemic Control (HbA1c) in the self-management of diabetes? A systematic review, Meta-analysis, and GRADE of 14 randomized trials. Diabetes Care. 2016;39:2089–95. 10.2337/dc16-0346. 27926892 10.2337/dc16-0346

[25] Quinn CC, Shardell MD, Terrin ML, et al. Mobile Diabetes intervention for Glycemic Control in 45- to 64-Year-old persons with type 2 diabetes. J Appl Gerontol. 2016;35:227–43. 10.1177/0733464814542611. 25098253 10.1177/0733464814542611

[26] Ryan EA, Holland J, Stroulia E, et al. Improved A1C levels in type 1 diabetes with Smartphone App Use. Can J Diabetes. 2017;41:33–40. 10.1016/j.jcjd.2016.06.001. 27570203 10.1016/j.jcjd.2016.06.001

[27] Holtz B, Mitchell KM, Holmstrom AJ, et al. An mhealth-based intervention for adolescents with type 1 diabetes and their parents: pilot feasibility and efficacy single-arm study. JMIR mHealth uHealth. 2021;9:e23916. 10.2196/23916. 34519670 10.2196/23916 PMC8479605

[28] Zhang L, He X, Shen Y, et al. Effectiveness of Smartphone App-Based Interactive Management on Glycemic Control in Chinese patients with poorly controlled diabetes: Randomized Controlled Trial. J Med Internet Res. 2019;21:e15401. 10.2196/15401. 31815677 10.2196/15401 PMC6928697

[29] Yu Y, Yan Q, Li H, et al. Effects of mobile phone application combined with or without self-monitoring of blood glucose on glycemic control in patients with diabetes: a randomized controlled trial. J Diabetes Investig. 2019;10:1365–71. 10.1111/jdi.13031. 30815973 10.1111/jdi.13031 PMC6717828

[30] Chhanabhai P, Holt A. Consumers are ready to accept the transition to online and electronic records if they can be assured of the security measures. MedGenMed. 2007;9:8. 17435617 PMC1924980

[31] Kumar P, Lee HJ. Security issues in healthcare applications using wireless medical sensor networks: a survey. Sens (Basel). 2012;12:55–91. 10.3390/s120100055. 10.3390/s120100055 PMC3279202 22368458

[32] Burnes D, Henderson CJ, Sheppard C, et al. Prevalence of Financial Fraud and scams among older adults in the United States: a systematic review and Meta-analysis. Am J Public Health. 2017;107:e13–21. 10.2105/AJPH.2017.303821. 28640686 10.2105/AJPH.2017.303821 PMC5508139

[33] Kim J, Park HA. Development of a health information technology acceptance model using consumers’ health behavior intention. J Med Internet Res. 2012;14:e133. 10.2196/jmir.2143. 23026508 10.2196/jmir.2143 PMC3510715

[34] Jeffrey B, Bagala M, Creighton A, et al. Mobile phone applications and their use in the self-management of type 2 diabetes Mellitus: a qualitative study among app users and non-app users. Diabetol Metab Syndr. 2019;11:84. 10.1186/s13098-019-0480-4. 31636719 10.1186/s13098-019-0480-4 PMC6794726

[35] Mayberry LS, Osborn CY. Family involvement is helpful and harmful to patients’ self-care and glycemic control. Patient Educ Couns. 2014;97:418–25. 10.1016/j.pec.2014.09.011. 25282327 10.1016/j.pec.2014.09.011 PMC4254324

[36] Henry SL, Rook KS, Stephens MA, et al. Spousal undermining of older diabetic patients’ disease management. J Health Psychol. 2013;18:1550–61. 10.1177/1359105312465913. 23325381 10.1177/1359105312465913 PMC4506743

[37] Mayberry LS, Rothman RL, Osborn CY. Family members’ obstructive behaviors appear to be more harmful among adults with type 2 diabetes and limited health literacy. J HEALTH COMMUNICATION. 2014;19(Suppl 2):132–43. 10.1080/10810730.2014.938840. 25315589 10.1080/10810730.2014.938840 PMC4199389

[38] Bennich BB, Røder ME, Overgaard D, et al. Supportive and non-supportive interactions in families with a type 2 diabetes patient: an integrative review. Diabetol Metab Syndr. 2017;9:57. 10.1186/s13098-017-0256-7. 28736580 10.1186/s13098-017-0256-7 PMC5521150

[39] Mayberry LS, Osborn CY. Family support, medication adherence, and glycemic control among adults with type 2 diabetes. Diabetes Care. 2012;35:1239–45. 10.2337/dc11-2103. 22538012 10.2337/dc11-2103 PMC3357235

[40] Dzerounian J, Pirrie M, AlShenaiber L, et al. Health knowledge and self-efficacy to make health behaviour changes: a survey of older adults living in Ontario social housing. BMC Geriatr. 2022;22:473. 10.1186/s12877-022-03116-1. 35650537 10.1186/s12877-022-03116-1 PMC9158350

[41] Ong WM, Chua SS, Ng CJ. Barriers and facilitators to self-monitoring of blood glucose in people with type 2 diabetes using insulin: a qualitative study. Patient Prefer Adherence. 2014;8:237–46. 10.2147/PPA.S57567. 24627628 10.2147/PPA.S57567 PMC3931581

