# Supplementary material for: Role perceptions and experiences of adult children in remote glucose management for older parents with type 2 diabetes mellitus: a qualitative study
Source: BMC Geriatr. 2024 Aug 3;24:653. doi: 10.1186/s12877-024-05224-6 (PMC11297597; doi:10.1186/s12877-024-05224-6)
Supplement: Supplementary file 1 — Supplementary Material 1 [file 12877_2024_5224_MOESM1_ESM.docx]

**Supplementary Table 1**. Quotes from interviewees

| Items | Themes | Sub-themes | Quotes |
| --- | --- | --- | --- |
| Role perception | Health decision- maker |  | N7: “As soon as I received the message that his blood sugar was 3.2 mmol/L, I called him to drink sugar water and test his blood sugar again 15 minutes later. I reduced his bedtime insulin by 4 units after consulting with the doctor. After that, he rarely suffered from hypoglycemia.” |
|  |  |  | N15: “The mobile app revealed that the adherence rate for blood sugar control was only 50%. The reason for blood sugar levels not meeting the standard may be related to excessive rice consumption. Therefore, I suggested she measure postprandial blood sugar, which was 16 mmol/L. Following the dietitian's advice, I recommended she limit her rice intake to 75g per meal. As she was unsure how to measure the rice, I bought an electronic scale and taught her how to use it to control her staple food intake. Consequently, her adherence rate improved from 50% to 60%.” |
|  | Remote supervisor |  | N1: “If he hadn't checked his blood sugar for a few days, I reminded him to check it.” |
|  |  |  | N7: “Although my father doesn’t live with me, I can keep an eye on his blood sugar through the mobile app. When his blood sugar is lower than 5.6 mmol/L, I remind him to eat 3 pieces of soda crackers or drink a glass of milk.” |
|  |  |  | N9: “As my father is old and has a bad memory, he would miss his medication. I checked to see if his blood sugar was up to standard via the mobile app and reminded him daily not to miss his medication.” |
|  | Health educators |  | N2: “I can check his blood sugar data anytime and anywhere through the app. One day he ate plain congee resulting in high blood sugar. Then I told him not to drink plain congee. If he wants it, he should add beans, wheat, and lean meat to plain congee.” |
|  |  |  | N4: “I received a message which showed that she suffered from hypoglycemia. It turned out that exercising on an empty stomach caused her hypoglycemia. I stressed the precautions to be taken in exercising. After that, she never exercises on an empty stomach.” |
|  |  |  | N13: “I explained how to use the medication, how it works and side effects.” |
|  | Emotional supporter |  | N1: “I saw his blood sugar fluctuating wildly via the mobile app and called him to ask why. He said he had poor sleep and wondered if he was suffering from depression. I comforted him that everything would be fine. After two weeks of adjustments, his mood was improved.” |
|  |  |  | N6: “My mother had anxiety and was terrified of hypoglycemia. I told her to take it easy and monitor her blood sugar regularly so that I could keep track of her blood sugar. I taught her ways to cope with hypoglycemia. She’s not so scared anymore and confident in her blood sugar control.” |
| Experiences of participation | Facilitators to participation | Convenience | N7: “Although my father doesn’t live with me, I can keep an eye on his blood sugar anytime and anywhere through the mobile app.” |
|  |  |  | N8: “I used to have to keep track of his blood sugar through his paper records. But now I can check his blood sugar through the app. It's very convenient.” |
|  |  |  | N9: “I used the mobile app to check that his blood sugar was up to standard. It's convenient.” |
|  |  | App user-friendly | N1: “The blood sugar data are presented in a visual curve, which makes management more intuitive and accurate. So I find it very, very useful.” |
|  |  |  | N10: “This app shows the adherence rate of blood sugar control, which is quite good.” |
|  |  |  | N12: “Information collected can be presented in a variety of graphical outputs. It’s so easy and intuitive now that I no longer worry her about misremembering or missing records.” |
|  |  | Digital empowerment | N6: “From the time I told her blood sugar control goals and taught her how to check her adherence rate via the mobile app, she paid more attention to her blood sugar and monitored blood sugar regularly.” |
|  |  |  | N14: “I learned that exercising after meal can lower postprandial blood sugar through the app. Based on her exercise and blood sugar, I worked with her on an exercise programme which increased her adherence rate for blood sugar control to 70%.” |
|  |  | Family-based health promotion | N8: “He has diabetic nephropathy. In order to control his protein intake and blood sugar, I help him prepare breakfast, lunch and dinner. The mobile app showed that his adherence rate has increased from 50% to 80%.” |
|  |  |  | N13: “I and my sister kept track of her blood sugar via the app and bought her medication monthly. After a period of medication, her blood sugar was under control.” |
|  | Barriers to participation | Privacy and security concerns | N5: “While we can help the elderly manage their blood sugar through mobile apps, I am worried that my mother’s information will be leaked.” |
|  |  |  | N7: “Nowadays there are a lot of scammers. The elderly are easily deceived. I am afraid that the personal information which is entered into the app will be utilized by an illegal actor after it is leaked.” |
|  |  |  | N15: “Is the information we enter into the app secure?” |
|  |  |  | N16: “I prefer other brands of blood sugar meters that do not access the Internet. Then I don't have to worry about information leaks.” |
|  |  | Adaptation and learning | N3: “It was hard for me to work on the app on my own at first.” |
|  |  |  | N8: “I wasn’t used to use the app at first.” |
|  |  |  | N11: “After 2 weeks of adaptation and consulting with doctors and nurses, I am now proficient in using the mobile app.” |
|  |  | Lack of knowledge about glucose management | N3: “I didn’t know his blood sugar control goals. There’s no point in focusing on his blood sugar via app.” |
|  |  |  | N10: “There are also a lot of diabetes-related videos that I should learn about. I'm not educated. This is a great opportunity.” |
|  |  |  | N11: “One day his blood sugar was 3.8 mmol/L. I was afraid that he would have hypoglycemic symptoms and told him not to take his pre-breakfast insulin injection. As a result, his blood sugar was 18 mmol/L in the 2 h after the meal. When an emergency occurs, I still don’t know what to do.” |
|  |  | Concerns oven the cost of test strips | N4: “The test strips are too expensive. I have already bought her test strips twice, which were not reimbursable. ” |
|  |  |  | N5: “I prefer to buy other brands of test strips online, it's cheaper.” |
|  |  |  | N9: “Do you provide complimentary test strips? He hesitates to incur expenses, thus rarely monitors his blood sugar. Consequently, I can access only limited data regarding his blood sugar levels.” |
|  |  |  | N12: “Do you offer free test strips?” |
|  |  |  | N16: “The test strips are so expensive that I can’t afford them.” |
